# Total knee arthroplasty revision risks depending on the bone cement used—Data from 50,545 knee replacements of the Catalan Arthroplasty Registry

**DOI:** 10.1002/jeo2.70271

**Published:** 2025-05-26

**Authors:** Daniel Perez‐Prieto, Katharina Koetter, Albert Fontanellas‐Fes, Olga Martínez‐Cruz, the RACat working group

**Affiliations:** ^1^ Ortophedic Surgery, Hospital del Mar Universitat Autònoma de Barcelona Barcelona Spain; ^2^ Department of Health Economics & Outcome Research Heraeus Medical GmbH Wehrheim Germany; ^3^ Departament de Salut Data Analytics Program for Research and Innovation in Health (PADRIS) Agència de Qualitat i Avaluació Sanitàries de Catalunya (AQuAS) Barcelona Spain

**Keywords:** antibiotic‐loaded bone cement (ALBC), bone cement, Catalan Arthroplasty Register (RACat), prosthetic joint infection (PJI), total knee arthroplasty (TKA)

## Abstract

**Purpose:**

Clinical data on individual bone cement brands and viscosities in cemented total knee arthroplasty (TKA) is scarce. The Catalan arthroplasty registry (RACat) documents usage of cement brands including viscosities and the inclusion of antibiotics. The objective was to compare the clinical performance of the widely used bone cement brand PALACOS® to other blinded bone cement brands in TKA using data from the RACat.

**Methods:**

Patient data on 50,545 primary TKA between 2007 and 2017 in the RACat were analysed retrospectively. Implant survival of PALACOS bone cement was compared to other blinded bone cement brand groups using the all‐cause revision risk as primary study endpoint.

**Results:**

Comparing implant survival, it was found that (1) PALACOS® (with or without gentamicin) was associated with a significantly lower revision risk compared to other cement brands (with or without antibiotics) (*p* = 0.001): RR PALACOS 2.03% versus RR other brands 3.88%, and RR PALACOS+G 1.84% versus RR other antibiotic‐loaded bone cements (ALBC) 3.85%; (2) ALBC (all brands) did not reduce the risk of revisions (*p* = n.s) compared to plain bone cements (PBC); and (3) the medium viscosity PALACOS MV+G showed the lowest reoperation risk versus other ALBC (all viscosities): RR 1.12% versus RR 3.85%. Competing‐risk regression models confirmed reduction in revision risk for all PALACOS compared with other brands (*p* = 0.001) and for PALACOS+G compared with other ALBC (*p* < 0.001) but showed no difference when comparing all ALBC with all PBC (*p* = 0.403). Comparing PALACOS MV+G with all other ALBC showed reduction of revision risk (*p* < 0.001) and no difference when comparing PALACOS medium viscosity (MV) with all other PBC (*p* = 0.108).

**Conclusions:**

Type of cement brand, viscosity and the addition of antibiotics have an impact on revision risk of TKA. Medium viscosity cement with gentamicin (PALACOS MV+G), for which no clinical data were previously available, was associated with the lowest revision risk in TKA.

**Level of Evidence:**

Level III, retrospective comparative study.

**Abbreviations**
ALBC: antibiotic‐loaded bone cements
AQuAS: Agency for Health Quality and Assessment of Catalonia
H1: first hypotheses
H2: second hypotheses
H3: third hypotheses
IIN: individual identification number
MV: medium viscosity
PBC: plain bone cements
RACat: Catalan Arthroplasty Registry
sHR: sub distributional hazard ratios
TKA: total knee arthroplasty

## INTRODUCTION

Worldwide, the number of joint replacement surgeries is increasing as a consequence of ageing societies [18, 24]. Despite being one of the most successful medical treatments, complications following arthroplasty still occur. These include thrombosis, infections, periprosthetic fractures, dislocations, prosthesis loosening and others. The revision risk is one of the most important outcome measures of joint replacement indicating the survival probability of implants. Clinical studies and registry reports have shown that failure risks of less than 1% per year are nowadays standard for total knee arthroplasty (TKA) [10, 29, 33]. However, the results still show considerable variations owing to aspects such as implant design, surgeon experience, patient‐related factors or surgical technique used [13, 21, 27]. Despite growing interest in cementless TKA [14], the cemented fixation of implant components remains the preferred option for many knee surgeons because of the benefit of formation of an immediate strong mechanical interlock of the implant with bone [5]. There is increasing evidence mainly from in vitro studies that the technique of cementation as well as the quality and mechanical properties of the bone cement have an important impact on clinical outcomes of knee arthroplasty [12, 39]. A further advantage of the cemented fixation is the use of antibiotic‐loaded bone cement (ALBC) to release antibiotics into the joint cavity for infection prophylactic purposes [30, 32].

Bone cement is primarily supplied as a two‐component system consisting of the liquid methyl methacrylate monomer and the powder polymethyl methacrylate. Starter and initiator substances in the powder and the liquid catalyse the chemical polymerization. The differences between commercially available bone cement brands lie in the nature of copolymers in the powder, the amount of starter and initiator molecules, the type and amount of included antibiotics, and the technique of sterilization [19, 20]. Another important differentiator is the initial viscosity of the cement. This describes the resistance of the cement after mixing to flow. Bone cements of high, medium and low viscosity vary in their composition and are used for different indications. One key aspect of cement viscosity is its influence on the depth of penetration into spongious tissue. Several studies have demonstrated that cements of medium to high viscosity allow a penetration of 2–5 mm into the spongious tissue in combination with jet lavage of the bone tissue and cement pressurization [11]. This cement mantle thickness has been established as a minimum threshold for sufficient implant stability [7, 37]. By contrast, bone cement of low viscosity tends to flow deeper into the tissue but bears risk of triggering more heat necrotic damage and may require more bone tissue removal in case of revision arthroplasty [1].

There is evidence from hip arthroplasty registries that the viscosity and the brand of bone cement influences the revision risk of common implant systems [9, 23]. The Swedish Arthroplasty Registry found that among the most commonly used bone cements in Sweden in knee arthroplasty, PALACOS R+G was associated with the lowest revision risk for all reasons, excluding infection and loosening [36]. However, for cemented TKA such clinical outcome data is scarce, and the issue of the best cement viscosity is still controversially discussed with some surgeons preferring quicker working times of high viscous cements and others preferring more extended working times of medium to low viscous cements. Higher ambient temperatures during storage of the cements or during surgeries may further explain why medium viscous cements are relatively popular in some Southern European hospitals.

In general, registries are powerful tools providing important information on the outcomes of surgical techniques and/or medical devices in a ‘real life situation’. As it is a long‐established registry with high participation of centres (between 50 and 53) and high number of recorded procedures since its inception in 2005, the authors chose the Catalan Arthroplasty Register (RACat) for data analysis. The main objective was to extend the evidence on clinical performance of individual bone cement viscosities and brands within the indication of TKA for regions where usage of cement for implant fixation is high. PALACOS cement brand was set as reference for comparison with other bone cements brands based on previous observations that it is the most commonly used cement in international knee arthroplasty registries and the Norwegian and Swedish registries already reported superior clinical performance in hip prosthesis fixation in comparison to other commonly used bone cement brands [9, 17, 22, 23, 36]. Therefore, PALACOS type cements were unblinded in the dataset provided by the RACat. With this study, we aim to answer the following study hypotheses: (H1) Do all cements of the brand PALACOS (with or without gentamicin; all viscosities) reduce the all‐cause revision risk compared to other brands (PBC and ALBC) used in total knee arthroplasties? (H2) Do ALBCs of all brands reduce the risk of all‐cause revisions compared to PBC? (H3) Does PALACOS MV (medium viscosity; with or without gentamicin) reduce the all‐cause revision risk compared to PBC and ALBC of other brands used in TKA?

## MATERIALS AND METHODS

### Study design

This was a retrospective observational registry study on patients undergoing TKA between 1 January 2007 and 31 December 2017 which were reported to the RACat Registry [3]. This study was initiated by a surgeon‐triggered inquiry in 2021 and approved by Ethics Committee of Hospital del Mar and the Advisory Board of RACat with the number 2020/9121. No external funding was provided, and the study was conducted independently of any commercial influence.

### Setting

Catalonia is an autonomous region in Spain with a population of 7,543,825 as of 1 January 2018 [35]. The Catalan healthcare system provides universal coverage and access to public healthcare for all residents living in Catalonia.

### Participants

The procedures considered had at least one of the joint components specified as cemented by its manufacturer or, if former had incomplete information, cement references and surgical specification of implant fixation. In this study, data were recorded in the RACat according to the following ICD‐9 procedure codes: 81.54 for primary procedures and 81.55, 00.80, 00.81, 00.82, 00.83, 00.84 for revision procedures.

### Study size

**Figure 1:**
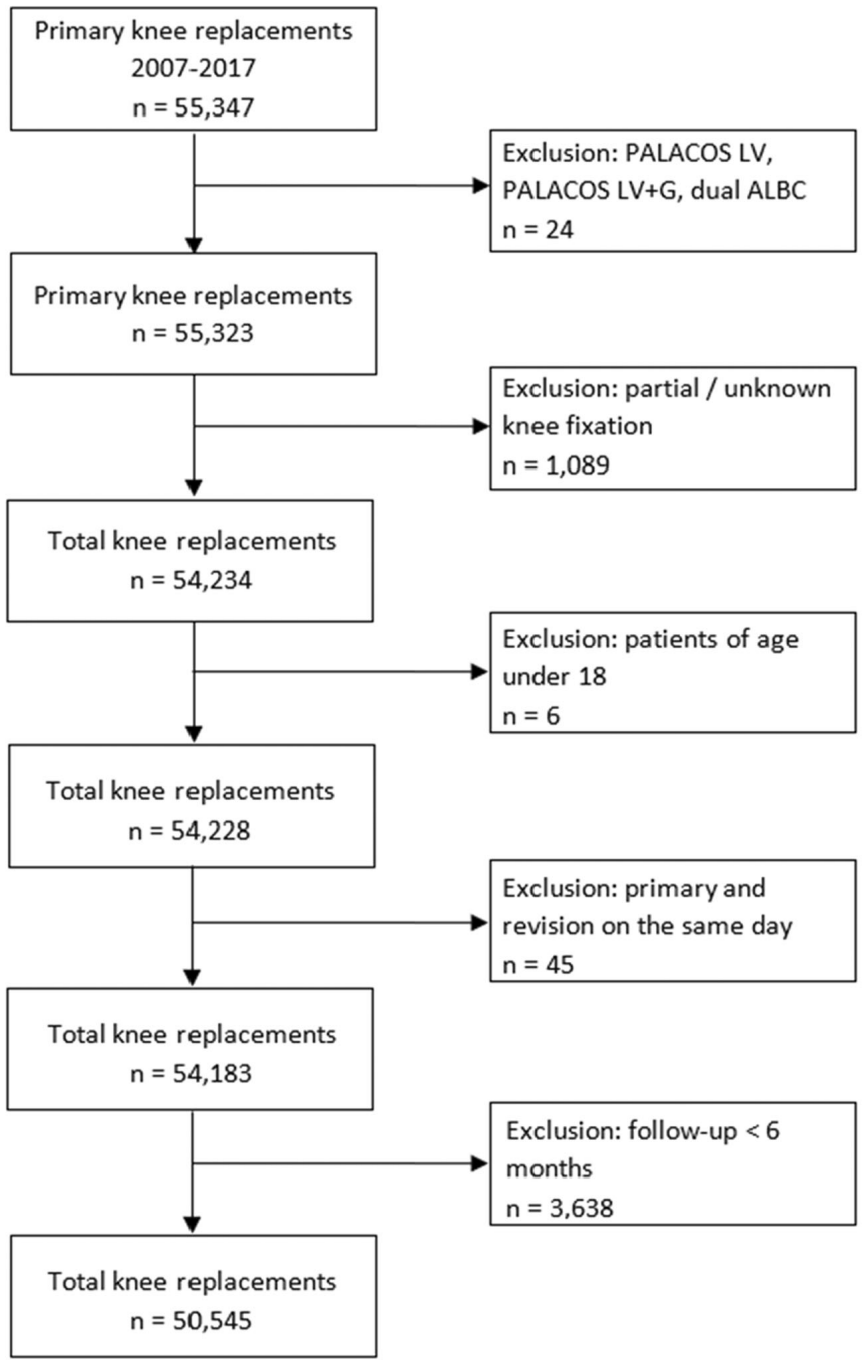
Flowchart of study population. 55,347 primary knee arthroplasty procedures were reported to the RACat between 2007 and 2017. Procedures using low‐viscosity PALACOS LV (PBC) and PALACOS LV+G (ALBC) were excluded due to insufficient sample size. Partial cemented or unknown fixation knee arthroplasties were also excluded. ALBC, antibiotic‐loaded bone cements; PBC, plain bone cements.

55,347 primary knee arthroplasty procedures were reported to the RACat between 2007 and 2017. We excluded procedures using low‐viscosity PALACOS LV (PBC) and PALACOS LV+G (ALBC) due to insufficient sample size for meaningful statistical analysis (Figure 1). Partial cemented or unknown fixated knee arthroplasties were excluded since this indication group would have required separate analyses and stratification along cement types would have yielded too small groups. Patients whose primary and revision surgery were dated on the same day or who were under the age of 18 at the time of surgery were excluded. At a minimum follow‐up at 6 months, patients being alive and revision‐free with follow‐up less than 6 months were excluded.

### Variables

**Figure 2:**
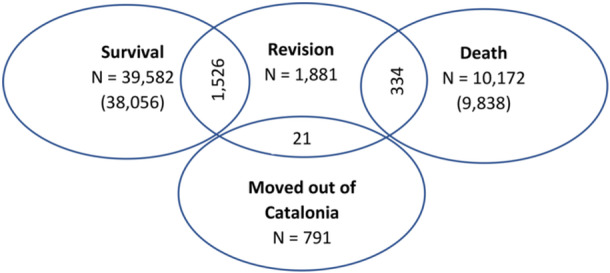
Distribution of survival, revision, death and relocation. Shows the distribution of patient outcomes (survival, revision, death and relocation) over the follow‐up period.

The all‐cause revision risk was set as the primary study endpoint. Revision was defined as a change of at least one implant component. PALACOS branded cements were unblinded whereas other cements allowed no brand identification, except viscosity and type of antibiotic contained (no antibiotics, gentamicin or tobramycin). Distribution of patients according to characteristics such as age, sex, number of comorbidities at time of admission, and fixation method (both latter were not universally collected) was tabulated in Table 1. Implant survival time and follow‐up time were calculated from surgical variables: date of primary surgery, surgery type, cement brand, implant type, surgical approach, date of revision surgery and living status. The distribution of indications for surgery was also recorded (Table 2). All participants were followed up starting from date of primary surgery until death, date of revision surgery, relocation or end of follow‐up (Figure 2).

**Table 1:** Overview of patient characteristics during the observation period 2007 and 2017 from RACat.

Variable	Total study population	ALBC	PBC	PALACOS	Other brands	Palacos+G	Other brands ALBC	Palacos MV	Palacos MV+G	Other brands PBC
Total	50,545	17,931	32,614	4341	46,204	1954	15,977	2195	1701	30,227
Revisions, *n* (%)	1881 (3.72)	651 (3.63)	1230 (3.77)	88 (2.03)	1793 (3.88)	36 (1.84)	615 (3.85)	46 (2.1)	19 (1.12)	1178 (3.9)
Death, *n* (%)	10,172 (20.12)	3639 (20.29)	6533 (20.03)	623 (14.35)	9549 (20.67)	286 (14.64)	3353 (20.99)	307 (13.99)	231 (13.58)	6196 (20.5)
Age, means (SD)	72.33 (7.64)	72.58 (7.79)	72.2 (7.56)	72.22 (7.54)	72.34 (7.65)	72.57 (7.36)	72.58 (7.84)	71.98 (7.77)	72.89 (7.34)	72.22 (7.55)
Female, *n* (%)	35,687 (70.6)	12,615 (70.35)	23,072 (70.74)	2994 (68.97)	32,693 (70.76)	1304 (66.73)	11,311 (70.8)	1550 (70.62)	1142 (67.14)	21,382 (70.74)
Comorbidity, mean (SD)	1.27 (1.11)	1.19 (1.11)	1.31 (1.11)	1.3 (1.14)	1.26 (1.11)	1.02 (1.09)	1.21 (1.11)	1.51 (1.12)	1.03 (1.1)	1.29 (1.1)
Unknown	790 (1.56)	270 (1.51)	520 (1.59)	115 (2.65)	675 (1.46)	71 (3.63)	199 (1.25)	40 (1.82)	46 (2.7)	476 (1.57)
Fixation method, *n* (%)										
Cemented	39,074 (77.31)	14,601 (81.43)	24,473 (75.04)	2555 (58.86)	36,519 (79.04)	1692 (86.59)	12,909 (80.8)	721 (32.85)	1490 (87.6)	23,610 (78.11)
Hybrid	6298 (12.46)	1499 (8.36)	4799 (14.71)	861 (19.83)	5437 (11.77)	34 (1.74)	1465 (9.17)	789 (35.95)	7 (0.41)	3972 (13.14)
Inverse hybrid	133 (0.26)	28 (0.16)	105 (0.32)	2 (0.05)	131 (0.28)	1 (0.05)	27 (0.17)	1 (0.05)	1 (0.06)	104 (0.34)
Unknown	5040 (9.97)	1803 (10.06)	3237 (9.93)	923 (21.26)	4117 (8.91)	227 (11.62)	1576 (9.86)	684 (31.16)	203 (11.93)	2541 (8,41)

*Note*: Describes demographic and clinical characteristics of the study population, including age, sex, comorbidities, fixation methods and distribution of bone cement types. Abbreviations: ALBC, antibiotic‐loaded bone cements; MV, medium viscosity; PBC, plain bone cements; RACat, Catalan Arthroplasty Registry.

**Table 2:** Distribution of indications for surgery.

Indication	*n*	%
Osteoarthritis	49,466	97.87
Osteoarthritis 2nd to acquired deformities	3655	7.23
Osteoarthritis and related disorders	45,808	90.63
Osteoarthritis postseptic arthritis	3	0.01
Other	1079	2.14
Postdysplastic arthrosis	32	0.06
Postnecrosis arthrosis	114	0.23
Posttraumatic arthrosis	114	0.23
Lower extremity fracture	43	0.09
Rheumatic disease	254	0.50
Avascular necrosis	60	0.12
Post‐traumatic necrosis	1	0.00
Neoplasia and/or tumours	22	0.04
Unknown	439	0.87

*Note*: Lists the primary indications for total knee arthroplasty in the study population, predominantly osteoarthritis.

### Statistical analyses

**Figure 3:**
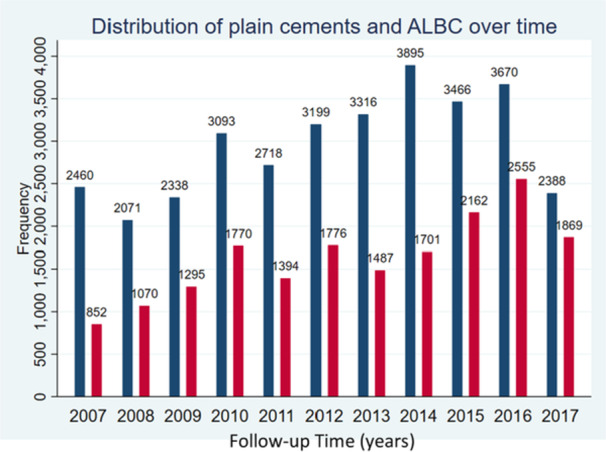
Distribution of plain (blue) cements and antibiotic‐loaded bone cements (ALBC) (red) over time. Shows the use of plain cements and ALBC by year during the observation period.

**Figure 4:**
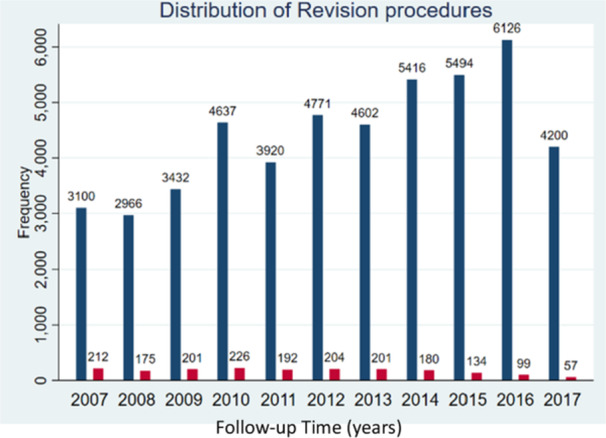
Distribution of primary (blue) and revision (red) procedures over time.

The study population was divided into five mutually exclusive comparison groups according to the bone cement used for fixation of the implant: all PALACOS versus all other brands (regardless of viscosity or antibiotics), all ALBC versus all plain bone cements (regardless of brands), PALACOS+G versus all other brands ALBC (regardless of viscosities and type of antibiotic), PALACOS MV+G versus all other brands ALBC (first of medium viscosity, last regardless of viscosities, both including antibiotics) and PALACOS MV versus all other PBC brands (first of medium viscosity, last regardless of viscosities, both no antibiotics). The total study population as well as patients per comparison group allocation were described by tabulating the distribution of patient characteristics, in frequency and percentage for categorical variables or mean and standard deviation for continuous variables (Table 1). Similarly, distribution of plain cement and ALBC use was displayed over time in Figure 3. Distribution of primary procedures and revision procedures over time was presented in Figure 4.

**Figure 5:**
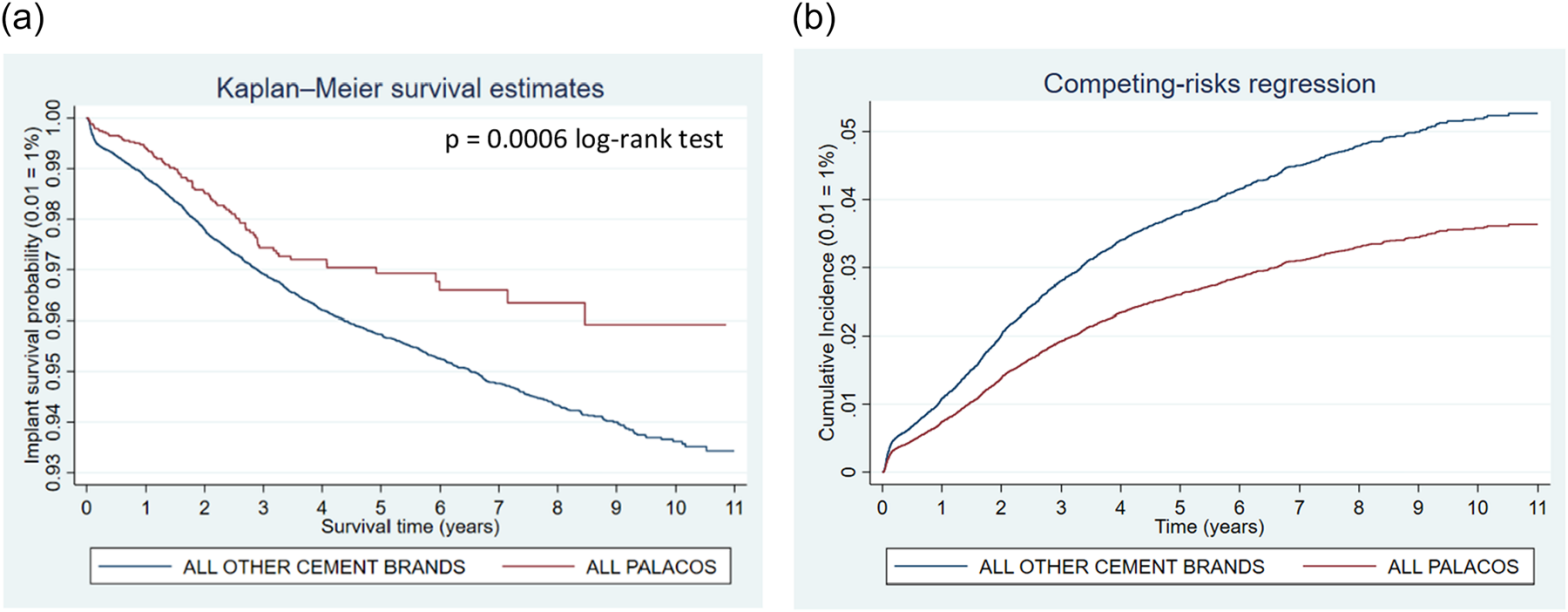
Comparison of revision risk of ALL PALACOS versus ALL OTHER CEMENT BRANDS. (a) Kaplan–Meier estimated survival curves for implant revision. (b) Competing‐risks regression resulting cumulative incidence curves for implant revision adjusted for age, sex and year cohort.

**Figure 6:**
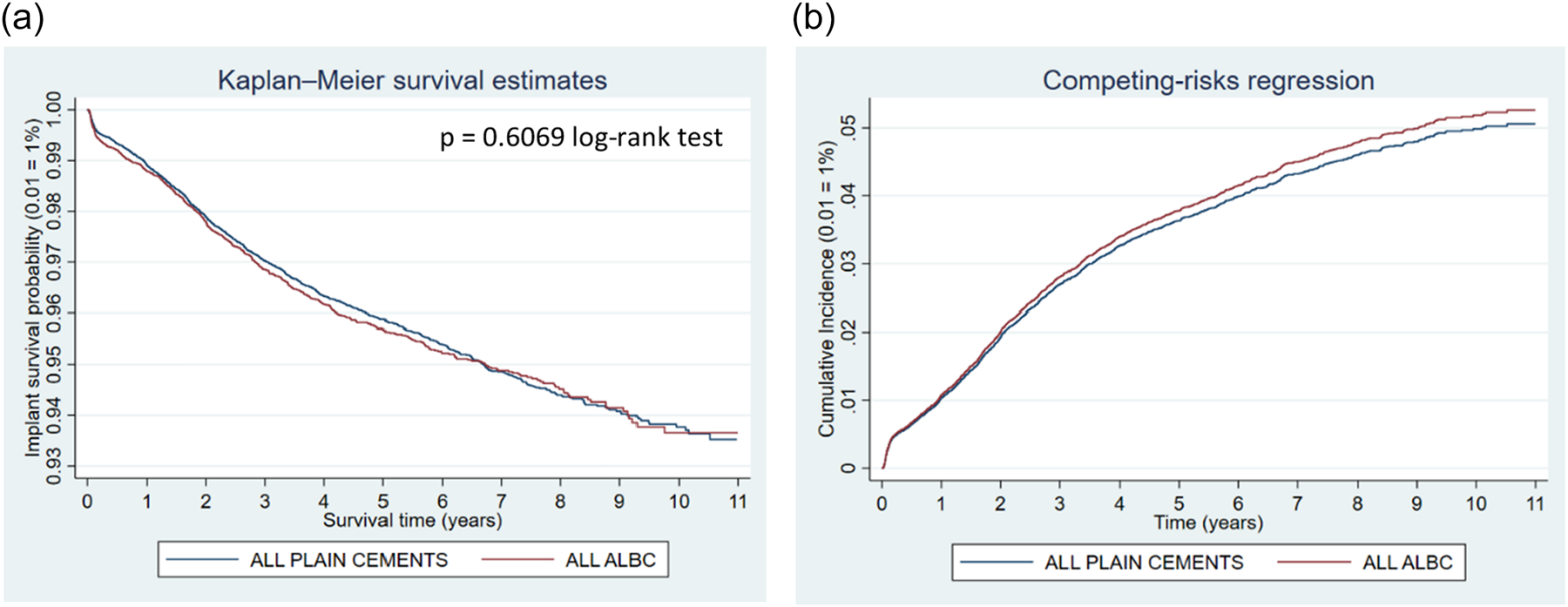
Comparison of revision risk of ALL ALBC versus ALL PLAIN CEMENTS. (a) Kaplan–Meier estimated survival curves for implant revision. (b) Competing‐risks regression resulting cumulative incidence curves for implant revision adjusted for age, sex and year cohort.

**Figure 7:**
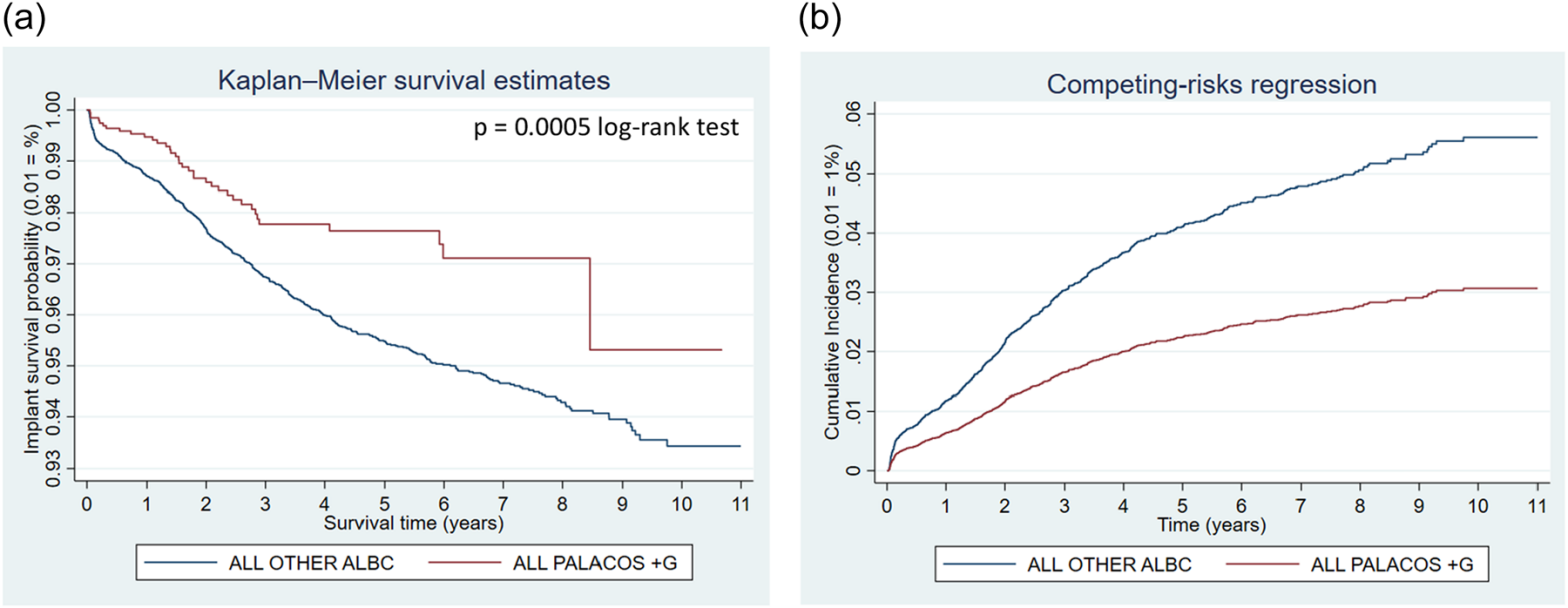
Comparison of revision risk of ALL PALACOS+G versus ALL OTHER antibiotic‐loaded bone cements (ALBC). (a) Kaplan–Meier estimated survival curves for implant revision. (b) Competing‐risks regression resulting cumulative incidence curves for implant revision adjusted for age, sex and year cohort.

**Figure 8:**
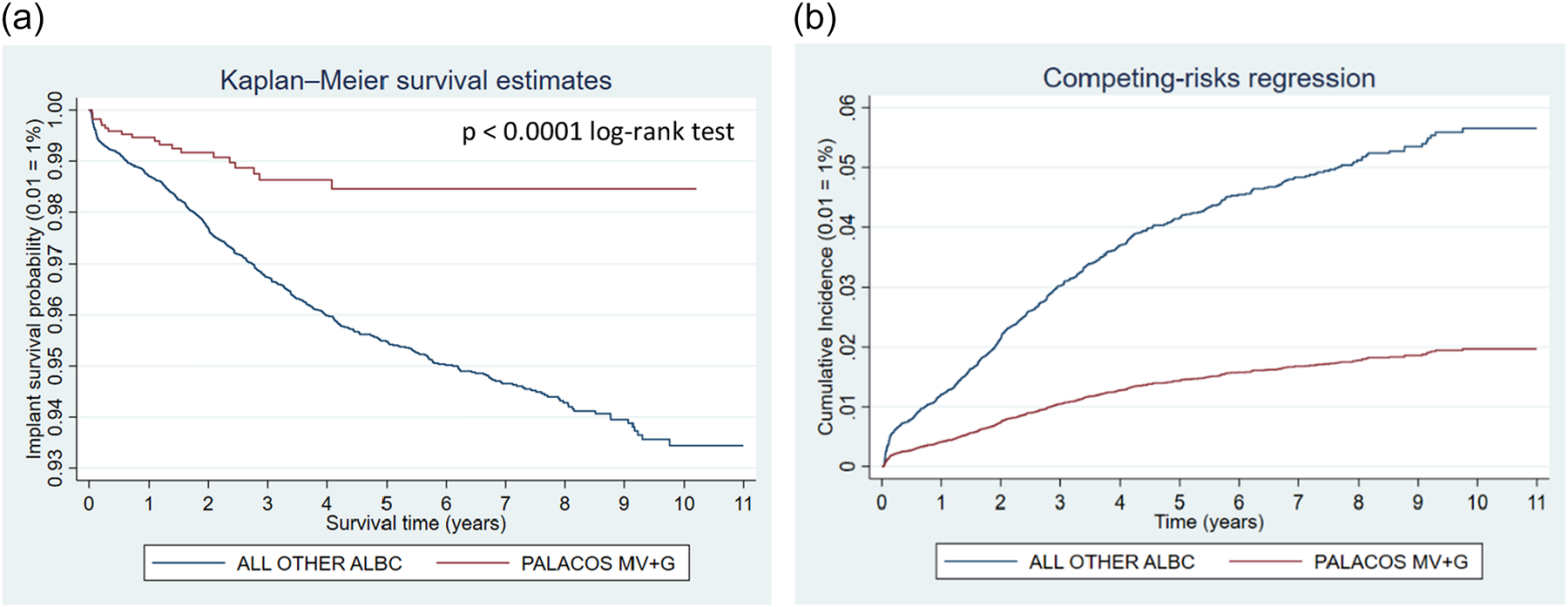
Comparison of revision risk of ALL PALACOS MV+G versus ALL OTHER antibiotic‐loaded bone cements (ALBC). (a) Kaplan–Meier estimated survival curves for implant revision. (b) Competing‐risks regression resulting cumulative incidence curves for implant revision adjusted for age, sex and year cohort.

**Figure 9:**
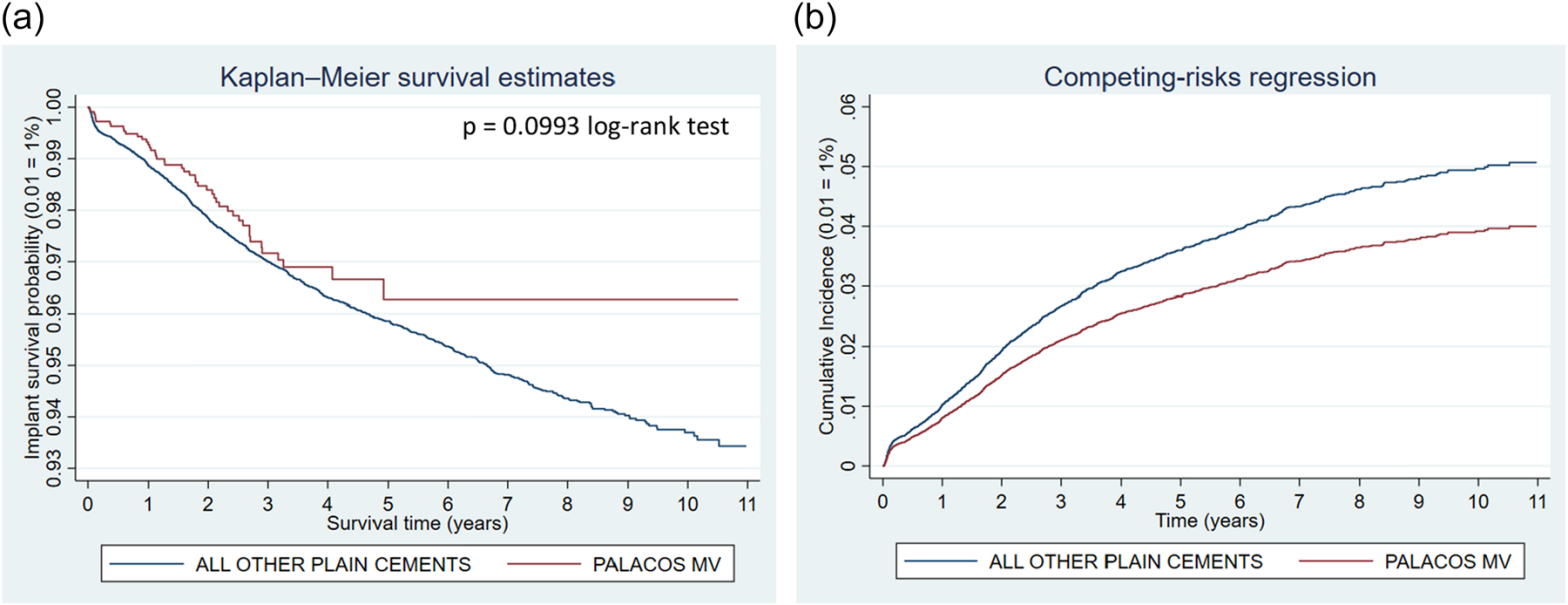
Comparison of revision risk of ALL PALACOS MV versus ALL OTHER PLAIN CEMENTS. (a) Kaplan–Meier estimated survival curves for implant revision. (b) Competing‐risks regression resulting cumulative incidence curves for implant revision adjusted for age, sex and year cohort.

The revision risk is shown in total numbers and percentages for all‐causes at 1 year, 5 years, complete follow‐up as well as for the major specified reasons for revision for complete follow‐up: infection, aseptic loosening, mechanic complications, prosthesis luxation and other reasons (Table 3). For each comparison group, log‐rank tests and Kaplan‐Meier plots comparing implant survival over time, cumulative incidence function curves and competing risk models were performed (Table 4; Figures 5, 6, 7, 8, 9). Besides the exclusion criteria, censoring was defined when there was no revision procedure registered as of follow‐up period end. Following a clinical approach, the researchers chose covariates for multivariable analyses that are known to be clinically relevant: age, sex and year cohort. While age, sex and year cohort were included as covariates, additional variables such as implant brand, surgical technique and hospital volume were not available in the dataset of RACat. These unmeasured factors could introduce residual confounding. The fit of cox proportional hazards models was tested using log‐log plots and Schoenfelds' residuals finding that proportional hazards assumption was violated in all models (*p* < 0.05). Results from competing‐risk models using death as competing event are presented with unadjusted and adjusted subdistributional hazard ratios (sHR), 95% confidence intervals (CIs) and corresponding *p*‐values (see Table 4). Cumulative incidence curves compare adjusted reoperation risks for the groups, using death as competing event. For all significance tests a level at 5% (*p* < 0.05) was chosen. The data were analysed using Stata 17.0 [34].

**Table 3:** Revision risk, by time points and overall, and by reason.

		Any cause, *n* (%)			Infection, *n* (%)	Aseptic loosening, *n* (%)	Mechanic compli cations, *n* (%)	Prosthesis luxation, *n* (%)	Other reasons, *n* (%)
Revision risk	*n*	1 year	5 years	All revisions					
Total	50,545	564 (1.12)	1631 (3.23)	1881 (3.72)	458 (0.91)	630 (1.25)	478 (0.95)	53 (0.1)	262 (0.52)
All PALACOS	4341	25 (0.58)	84 (1.94)	88 (2.03)	27 (0.62)	20 (0.46)	28 (0.65)	1 (0.02)	12 (0.28)
All other brands	46,204	539 (1.17)	1547 (3.35)	1793 (3.88)	431 (0.93)	610 (1.32)	450 (0.97)	52 (0.11)	250 (0.54)
All ALBC	17,931	212 (1.18)	578 (3.22)	651 (3.63)	174 (0.97)	192 (1.07)	182 (1.02)	26 (0.15)	77 (0.43)
All plain cements	32,614	352 (1.08)	1053 (3.23)	1230 (3.77)	284 (0.87)	438 (1.34)	296 (0.91)	27 (0.08)	185 (0.57)
All PALACOS+G	1954	10 (0.51)	33 (1.69)	36 (1.84)	12 (0.61)	9 (0.46)	13 (0.67)	1 (0.05)	1 (0.05)
All other brands ALBC	15,977	202 (1.26)	545 (3.41)	615 (3.85)	162 (1.01)	183 (1.15)	169 (1.06)	25 (0.16)	76 (0.48)
PALACOS MV+G	1701	9 (0.53)	18 (1.06)	19 (1.12)	11 (0.65)	0	8 (0.47)	0	0
PALACOS MV	2195	15 (0.68)	46 (2.1)	46 (2.1)	15 (0.68)	6 (0.27)	14 (0.64)	0	11 (0.5)
All other plain cement brands	30,227	337 (1.11)	1002 (3.31)	1178 (3.9)	269 (0.89)	427 (1.41)	281 (0.93)	27 (0.09)	174 (0.58)

*Note*: Summarizes revision rates at 1 year, 5 years and over the full observation period, broken down by specific causes such as infection, aseptic loosening, mechanical complications, prosthesis luxation and other reasons. Abbreviations: ALBC, antibiotic‐loaded bone cements; MV, medium viscosity.

**Table 4:** Fine‐Grey's competing‐risks regression subdistributional hazard ratios for revision.

Comparison groups	Unadjusted sHR (95% CI)	*p* value	Adjusted sHR (95% CI)	*p* value
All PALACOS	0.66 (0.54–0.82)	<0.001	0.68 (0.55–0.85)	0.001
All other brands	1 reference		1 reference	
All ALBC	1.01 (0.92–1.11)	0.796	1.04 (0.95–1.15)	0.403
All plain cements	1 reference		1 reference	
All PALACOS+G	0.54 (0.39–0.76)	<0.001	0.54 (0.38–0.76)	<0.001
All other brands ALBC	1 reference		1 reference	
PALACOS MV+G	0.34 (0.22–0.54)	<0.001	0.34 (0.22–0.54)	<0.001
All other brands ALBC	1 reference		1 reference	
PALACOS MV	0.74 (0.55–1.0)	0.047	0.79 (0.58–1.06)	0.108
All other plain cement brands	1 reference		1 reference	

*Note*: Presents unadjusted and adjusted subdistributional hazard ratios (sHR) comparing revision risks between different bone cement types and brands. Abbreviations: ALBC, antibiotic‐loaded bone cements; CI, confidence interval; MV, medium viscosity; sHR, subdistributional hazard ratios.

### Data sources

The RACat is a public health‐based population register collecting standardized data on hip and knee replacements in Catalonia, Spain. Only procedures that are publicly funded are reported [2]. The register is managed and coordinated by the Agency for Health Quality and Assessment of Catalonia (AQuAS) and reporting is performed digitally [2]. During the data collection time of this study, reporting to the RACat was not mandatory. In total, 53 out of 56 different health centres in the Integrated Public Healthcare System of Catalonia reported their procedures to the RACat, the proportion varying between years. 89% of all primary and 73% of all revision knee replacements in Catalonia are captured in the RACat between 2007 and 2017 [3].

### Data access, linkage and cleaning methods

The researchers had access to a dataset provided in Excel by the AQuAS [26]. With regards to data use and confidentiality, patient‐level information obtained from RACat was anonymized and de‐identified prior to the analysis. The data delivered already included the primary procedures linked with its corresponding revision procedure data. This dataset sourced its information from the RACat Catalogue, RACat Registry, Minimum Data Set at Hospital Discharge, and the Central Insurance Register. Data entries were linked prior to using before patients Individual Identification Number (IIN), centre, date of admission, joint and type of procedure (primary or revision).

## RESULTS

Of the total study population included in the analysis (*n* = 50,545), 1881 patients needed their implant to be revised (revision risk 3.72%) and 10,172 died (20.12%) during the observation period. The mean age was 72.33 years and 70.6% of patients were female. While the majority of patients had an unknown ASA score (77.86%), 15.24% had ASA score II and 6.07% had ASA score III. 27.9% of patients counted no comorbidity and 67.01% counted 1–3 comorbidities. 77.31% of the patients received a fully cemented, 12.46% a hybrid, and 0.26% an inverse hybrid knee implant. Posterior‐stabilized prostheses (46.14%) were predominantly implanted, followed by cruciate‐retaining (33.23%) and posterior cruciate‐substituting implants (16.46%). For further details see Table 1. Furthermore, the majority of the study population (97.87%) had some form of osteoarthritis as an underlying indication for surgery (Table 2).

## ALL PALACOS VERSUS ALL OTHER BONE CEMENT BRANDS

It was found that 2.03% of all knee prostheses cemented with PALACOS were revised in the observation period versus 3.88% when using a different bone cement (Table 3). The Kaplan–Meier curves as well as cumulative incidence graph confirm by showing diverging survival estimate curves of the two groups (Figure 5).

Comparing the two groups, the former showed lower risks in for all reasons for revision separately. Competing‐risks regression model accounting for death, results in adjusted sHR at 0.68 (95% CI: 0.55–0.85; *p* = 0.001) for all PALACOS compared to all other cement brands.

## ALL ALBC VERSUS ALL PLAIN CEMENTS

All‐cause revision risk was found at 3.63% for ALBC and 3.77% for plain cements (Table 3). Patients receiving ALBC were found to have higher risk of revision for infection, mechanical complications, prosthesis luxation, but lower risk of aseptic loosening and other reasons compared to patients receiving plain cements. Both the Kaplan–Meier curve and the cumulative incidence function indicate similar graph behaviour (Figure 6) as well as the competing‐risks regression model found adjusted sHR at 1.04 (95% CI: 0.95–1.15; *p* = 0.403) for all ALBC compared to all plain cements.

## ALL PALACOS+G VERSUS ALL OTHER ALBC BRANDS

From those patients receiving gentamicin containing PALACOS+G cement 1.84% of the knee implants had to be revised compared to 3.85% of those receiving any other commercial ALBC (Table 3). The PALACOS+G group showed a lower risk of revision for all reasons individually. Kaplan–Meier curves and cumulative incidence graphs (Figure 7) show diverging curves for the two comparison groups. Competing‐risks model resulted in adjusted sHR at 0.54 (95% CI: 0.38–0.76; *p* < 0.001) for all PALACOS+G compared to all other ALBC brands.

## PALACOS MV+G VERSUS ALL OTHER ALBC BRANDS

The revision risk for PALACOS MV+G cement was found at 1.12% versus 3.85% for all other ALBC brands (Table 3). PALACOS MV+G showed reduced risk of revision for each reason for revision individually. Both Kaplan–Meier curves and cumulative incidence graphs show significant differences between the two groups (Figure 8). The regression analyses demonstrated adjusted sHR at 0.34 (95% CI: 0.22–0.54; *p* < 0.001) when PALACOS MV+G was compared with all other ALBC brands.

## PALACOS MV PLAIN VERSUS ALL OTHER PLAIN CEMENT BRANDS

Plain cement PALACOS MV showed revision risk at 2.1% and all other plain cement brands at 3.9%. Comparison of reasons for revision yielded lower risks in each category for PALACOS MV (Table 3). Kaplan–Meier curves and cumulative incidence graphs indicate difference in revision risk, however, nonsignificantly (Figure 9). Competing‐risks regression models comparing PALACOS MV and all other plain cement brands demonstrated unadjusted sHR at 0.74 (95% CI: 0.55–1.0; *p* = 0.047) and adjusted sHR at 0.79 (95% CI: 0.58–0.1.06; *p* = 0.108).

## DISCUSSION

Our study suggests that bone cement brand, viscosity and the addition of gentamicin may influence revision rates in TKA.

We have chosen the RACat Registry for analysis as it is together with the Norwegian and Swedish arthroplasty registries one of few such databases collecting information on the specific brand of bone cement used for the fixation of the prosthesis. Because of the already reported superiority of the PALACOS‐type bone cements in the national hip Registry of Norway, Sweden, and the combined database of the Nordic Arthroplasty Register Association the request to the RACat was designed in this way that the revision risks of all knee prostheses cemented with PALACOS‐type cements were compared with the blinded data of other bone cements brands [9, 17, 23, 36]. Although the comparison of these groups is relatively heterogenic, this study rationale is justified according to the research hypotheses of the present study.

As for the implant failure observed for all PALACOS‐type bone cements compared to the group of other commonly used cements recorded in the RACat, a significantly reduced revision risk by 32% was found in the adjusted analyses for all those TKA in which PALACOS was used for implant fixation, regardless of cement viscosity and antibiotic load. These observations acknowledge the clinically superior performance of this cement brand in the field of TKA. This finding was confirmed when PALACOS+G was found to reduce revision risk by 46% in adjusted competing‐risks analysis when compared to all other ALBC brands.

The comparison of revision risk between PBC and ALBC without further consideration of a specific cement brand did not yield significant differences. These results confirm what has been previously reported by the Australian Registry or by clinical studies from Canada and the United States but contradict the findings from the Nordic Registries and from the United Kingdom‐based NJR [4, 6, 8, 15, 16, 28]. In the latter registries it was found that the presence of antibiotics in commercial bone cement was associated with a lower number of revisions. The reasons for these opposed observations are not clear. However, it may be speculated that varying usage rates of specific ALBC brands between the territories combined with differences in antibiotic elution capacities influence the results. Indeed, significant brand‐specific differences in the release of antibiotics from the cement matrix leading to higher or lower antimicrobial efficacies have been observed in several laboratory studies [25, 38]. Another explanation could be the different rationales of use of ALBC being standard in most of the cemented routine arthroplasties in the Nordic countries and the United Kingdom versus their differential patient‐risk stratified usage in the United States or in Canada because of the stricter regulatory approval status there [31]. The reduced risk of revisions due to infection and overall (all reasons) in groups all PALACOS+G and PALACOS MV+G may argue in this direction on the background of its observed antibiotic elution properties [25, 38].

Another important finding of our analysis was the clinical safety of the PALACOS MV+G cement for which there is no data available. Prostheses fixated with this cement showed the lowest risk of failure in the competing‐risk models and Kaplan–Meier curves when compared to other ALBC brands with 66% risk reduction. This observation holds true for plain PALACOS MV (reduction of revision risk by 21% in the adjusted model) when compared to other plain cement brands as well. Since clinical outcome data for medium‐viscosity cement have not been published to our knowledge so far, these observations provide evidence for its use in TKA.

While analyses were based on real‐world evidence, some study limitations need to be addressed. In general, selection bias in a cohort study occurs as a consequence of loss to follow‐up. In our cohort, the number of excluded patients due to reasons listed in the methods section was 8.68% of the initial study size. Furthermore, it is important to note that reporting to the RACat only became mandatory in 2017 and, therefore, not all procedures are documented in the database. However, the completeness of revision procedures during the period from 2007 to 2017 was calculated to be at 73% by the RACat database system [3]. In addition, only publicly funded procedures are reported to the RACat whereby privately funded procedures were not included in the analyses. Another bias is the fact that real‐world data was used which does not allow for perfect factor identification as not all variables can be held constant like in a clinical trial setting. For example, surgeon's personal cementing technique preferences and cement mixing systems may have influenced the outcomes. In addition, we were not able to relate the performance of the cement with individual prosthesis designs, coatings and implant brands. This study is limited by potential selection bias, as surgeons may have preferred ALBC in higher‐risk patients. Additionally, implant brand, cementation technique and surgical approach were not controlled for, which may introduce residual confounding. Additionally, the implant brand, surgical technique, or cementation method were not controlled for, which could introduce residual confounding. Future studies should incorporate these factors for a more comprehensive analysis.

## CONCLUSION

Analysis of the RACat data demonstrates that the type of cement brand, the viscosity and the addition of antibiotics have an impact on revision risk of TKA.

It was confirmed (1) that PALACOS branded cements led to significantly lower all‐cause revision risk than the average of all other cement brands documented in the Registry, (2) that the revision risks between ALBC and plain cement were not statistically different without further consideration of the cement brand and (3) that the medium viscosity cement with gentamicin (PALACOS MV+G) for which no clinical data were previously available was associated with the lowest revision risk in TKA.

Tracks the number of primary and revision TKA procedures performed each year.

## The RACat WORKING GROUP

Mireia Espallargues Carreras, Network for Research on Chronicity, Primary Care and Health Promotion (RICAPPS). Francesc Pallisó Folch, Orthopaedic Surgery Service, University Hospital Santa María, Lleida, Spain.

## AUTHOR CONTRIBUTIONS

**Daniel Pérez‐Prieto**: Critical manuscript review; study design; data interpretation; study supervision. **Katharina Koetter**: Study design; data collection; literature review; manuscript writing. **Albert Fontanellas‐Fes**: Critical manuscript review; study design; data interpretation. **Olga Martinez‐Cruz** and the **RACat working group** contributed by providing and facilitating access to the arthroplasty registry data, and offered support for its analysis.

## CONFLICT OF INTEREST STATEMENT

Katharina Koetter is an employee of Heraeus Medical GmbH, the manufacturer of Palacos cement, which is discussed in the study. The remaining authors declare no conflicts of interest.

## ETHICS STATEMENT

The study protocol was reviewed and approved by the Ethics Committee of *Comité Ético de Investigación con Medicamentos (CEIM) grupo hospital Quirónsalud‐Catalunya* with the approval number 2019/91‐COT‐DEX. Informed consent was obtained from all participants in this study, and the ethical principles of the Declaration of Helsinki were adhered to.

## Data Availability

The data used in this study were obtained from the RACat Registry by a surgeon‐triggered inquiry in 2021 and approved by Ethics Committee of Parc Salut Mar and the Advisory Board of RACat with the number 2020/9121. Due to privacy and confidentiality agreements, the raw dataset is not publicly available. Access to anonymized data were granted upon reasonable request to the corresponding author.
